# Severe hypokalemia secondary to abuse of β-adrenergic agonists in a pediatric patient: Case report

**DOI:** 10.1590/2175-8239-JBN-2019-0020

**Published:** 2019-05-30

**Authors:** Carolt Arana Aliaga, Leonor Fayos de Arizon, Rosario Montañés Bermúdez, Jose A. Ballarín Castán, Anna Vila Santandreu

**Affiliations:** 1 Nephology Service, Fundació Puigvert, Barcelona, España.; 2 Laboratory Service, Fundació Puigvert, Barcelona, España.

**Keywords:** Hypokalemia, Factitious Disorders, Adrenergic beta-Agonists, Albuterol, Terbutalin, Hipopotassemia, Transtornos Autoinduzidos, Agonistas Adrenérgicos beta, Albuterol, Terbutalina

## Abstract

This study reports a case of a 13-year-old male with a 3-year history of severe and intermittent hypokalemia episodes of unknown origin, requiring admission to the intensive care unit (ICU) for long QT syndrome (LQTS), finally diagnosed of redistributive hypokalemia secondary to the abuse of β-adrenergic agonists in the context of a probable factitious disorder.

## RESUMO

O presente estudo relata o caso de um jovem de 13 anos de idade com histórico, há três anos, de episódios de hipocalemia grave intermitente de origem desconhecida, internado em unidade de terapia intensiva (UTI) por síndrome do QT longo (SQTL). O paciente foi diagnosticado com hipocalemia por redistribuição secundária ao abuso de agonistas β-adrenérgicos, em contexto de provável transtorno factício.

## INTRODUCTION

Hypokalemia is one of the most frequent hydroelectrolytic disorders in children. It is associated with renal, cardiac, respiratory, and digestive pathologies, and can cause life-threatening disturbances such as respiratory failure, cardiac arrhythmias, and cardiac arrest^1^. In the pediatric population, gastrointestinal and urinary losses of potassium are the most frequent causes^2^ while pharmacological causes are rare. Among the drugs related to hypokalemia are the ones that increase potassium intracellular uptake (beta adrenergic agents, barium, and antipsychotic) and those that increase urinary losses, mainly diuretics^2^^,^ 4.

Here we describe the case of a 13-year-old male with a 3-year history of severe and intermittent episodes of hypokalemia of unknown origin. He was admitted to our center for the first time after a sudden episode of severe symptomatic hypokalemia, and a diagnosis of redistributive hypokalemia secondary to the intake of β-adrenergic drugs, probably associated with a factitious syndrome, was made.

## CASE PRESENTATION

An apparently healthy 13-year-old male was admitted in the emergency department with symptoms of palpitations, muscle pain, tremors, and headache of 3-h duration. He denied fever, diarrhea, vomiting, or ingestion of any kind of drug. On physical examination, the patient had a good general condition, with height and weight on the 50^th^ percentiles for age and gender. He was euvolemic, pale, and sweating. Cardiac auscultation revealed the presence of rhythmic tachycardia without murmurs or friction, and there was tachypnea without added bruises. He also presented weakness of the lower limbs and distal hand tremor. There were no skin lesions or edema.

Multiple medical results and 16 discharge reports from different hospitals were provided by the parents. The current clinical symptoms started when he was 10 years old and since that time he had had many recurrent episodes of severe hypokalemia that had led to frequent hospital admissions, one of them in the intensive care unit due to long QT syndrome (LQTS). Many diagnoses had been ruled out, e.g. familial hypokalemic periodic paralysis, by specific genetic testing.

The patient had a medical history of previous episodes of allergic asthma with sporadic use of terbutaline, gastritis due to *H. pylori*, sinus tachycardia, surgical correction of strabismus, appendicectomy, and a high-flow priapism secondary to arteriovenous fistula that had been treated with selective arterial embolization when he was 6 years old. Importantly, the medical team was informed that some years previously, as a consequence of unexplained detection of benzodiazepines in the patient’s blood, custody of the child had been temporarily withdrawn from the mother.

Amazingly, the minor behaved as an adult. He had the role of interlocutor with the medical team and his comments were full of medical vocabulary that showed technical knowledge that did not correlate well with his age.

Blood analysis at admission showed the following values: hemoglobin 12.3 g/dL, leukocytes 11.68×10^9^/L, sodium 142 mmol/L, potassium 2.4 mmol/L, chloride 101 mmol/L, urea 4 mmol/L, creatinine 53 µmol/L, glomerular filtration rate (Schwartz formula) 112 mL/min/1.73 m^2^, glucose 5.6 mmol/L, transtubular potassium gradient 4.9, venous blood acid-base equilibrium pH 7.33, and HCO_3_^-^ 24.5 mmol/L. Urinalysis showed pH 6.5, sodium 162 mmol/L, potassium 38 mmol/L, urea 388 mmol/L, creatinine 22.8 mmol/L, urine osmolality 982 mOsm/kg, and fractional potassium excretion 36.8%. Proteinuria and hematuria were not observed. Hormone analysis revealed aldosterone after 30 min rest of 0.06 nmol/L and urinary aldosterone of 7.0 nmol/24 h. ECG showed sinus tachycardia of 120 bpm and a QT interval of 460 ms (max 440 ms). Renal ultrasound was normal and did not show lithiasis or nephrocalcinosis.

The treatment strategy consisted in intravenous potassium replacement, reaching normal potassium plasma levels after 12 h of parenteral infusion, with no need for further potassium supplements to maintain stable potassium levels in blood after discontinuation of parenteral infusion.

Two days after admission the patient was not receiving any potassium supplement or pharmacological treatment. He was asymptomatic and had potassium levels of 4.4 mmol/L in his 8:45 a.m. blood test. Suddenly he complained of intense headache, distal tremor, and tachycardia, and a second blood test performed at 2:15 p.m. revealed that the serum potassium level had decreased to 3.4 mmol/L. Thereafter, over a 2-h interval and without any specific treatment, the symptoms gradually disappeared and the patient was then discharged with oral potassium supplements.

Given the combination of transient and recurrent severe hypokalemia and rapid response to first-line treatment, as well as the presence of clinical signs suggestive of adrenergic crisis, a possible overdose of β-adrenergic agonist drugs was suspected. The two blood samples obtained on the day of discharge were analyzed at the Catalonian Antidoping Laboratory. Plasma salbutamol levels of 3 ng/mL and 65 ng/mL were present in the 8:45 a.m. and 14:15 p.m. blood samples, respectively [reference range for peak plasma concentration after 0.04-0.1 mg inhaler dose = 0.6-1.4 ng/mL^3^. These findings confirmed the diagnosis of hidden abuse of β-adrenergic agonists in the context of a possible factitious syndrome.

## DISCUSSION

Hypokalemia is defined as a plasma potassium level below 3.5 mmol/L. The related mortality risk increases significantly when plasma potassium levels fall below 2.5 mmol/L^4^. Hypokalemia is mainly related to three pathogenic mechanisms: extrarenal potassium wasting (usually gastrointestinal), which is the most frequent cause in children^2^; redistribution to the intracellular space; and renal potassium wasting^5^. It is a relatively common diagnosis in hospitalized pediatric patients, especially in critical care units^6^. Hypokalemia modifies the cell membrane polarization^7^, causing various clinical manifestations such as weakness, hyperreflexia, and hypotonia, all of which were present in our patient. The most severe disturbances due to hypokalemia affect the cardiovascular system^2^; Schaefer and Wolford described flattening of the T wave, the appearance of a U wave, and LQTS^8^. In the reported case, the patient had a previous history of LQTS that required admission to the ICU.

In our patient, gastrointestinal and renal potassium wasting was easily ruled out by anamnesis, tests, and history, and the associated clinical signs of tremors, tachycardia, headache, and sweating were highly suggestive of adrenergic crisis. The described symptoms are frequent side effects of β-adrenergic agonist overdose as a result of peripheral vascular dilatation^9^. On the other hand, the rapid and sustained normalization of serum potassium levels without the need for further oral supplements was also suggestive of a redistribution effect rather than a real potassium loss. Consequently, the diagnostic hypothesis was that our patient suffered hypokalemia due to redistribution secondary to the use of sympathomimetic bronchodilators. Analysis of the two blood samples, obtained pre- and post-crisis, confirmed our medical suspicion, although we could not know either the administered dose or the route of administration. The mechanism of action of adrenergic stimulants such as terbutaline and salbutamol is activation of adenyl cyclase, increasing the intracellular cyclic AMP, which stimulates the pump NA+/K+-ATPase and facilitates intracellular uptake of potassium^5^

Hoikka et al. reviewed the most frequent causes of poisoning in 334 children admitted to an emergency department and reported oral intake to be the most common route of administration and the β-adrenergic agonist terbutaline to be the most common poisoning substance (12.3%)^10^. The therapeutic dose of salbutamol varies between 0.3 and 0.8 mg/kg/day. Drug overdose has been reported when the dose exceeds 10-20 times this reference value^11^, and it is less frequent if the route of administration is by inhalation.

The management of hypokalemia depends on the potassium level and the presence of symptoms. When there are signs of cardiac conduction disturbance, it is recommended to start intravenous replacement using 0.5-1 mEq/kg (40-50 mEq/L) in saline with an infusion rate of 0.3-0.5 mEq/kg/h. If there is cardiac arrhythmia, the dose may be increased to 0.5-1 mEq/kg/h^2^. This therapeutic strategy was used in our patient, achieving normal potassium levels after 12 h of treatment.

In the reported case, significant clues in the investigation were the undiagnosed severe hypokalemia despite multiple hospital admissions, the presence of recurrent and sometimes severe clinical manifestations followed by asymptomatic periods, the handling of technical concepts and medical vocabulary by the child, and the previous deprivation of maternal custody due to presence of traces of benzodiazepine in the child’s blood.

The presence of salbutamol in the child’s blood in spite of the absence of a medical indication for its administration during hospital admission and the repeated denial of its use by the family and the child himself suggests that this is a factitious disorder. Munchausen syndrome is characterized by the presence of factitious symptoms repeatedly caused by the subject him- or herself, sometimes with physical self-harm to induce the appearance of these symptoms. This condition generally begins in adulthood and sometimes involves invasive diagnostic procedures and prolonged pharmacological treatment^12^^-^ 15. In the related psychiatric disorder commonly referred to as Munchausen syndrome by proxy, physical or psychological symptoms are imposed on another person (usually on children by parents) with the intention of deception and without any evident intention of benefitting from the abuse; this is termed “factitious disorder imposed on other”^16^. It is not considered a pediatric disorder but the external manifestation of an adult psychiatric disease^15^^,^ 17. However, in teenagers, whose autonomy makes them capable of taking personal decisions and of self-awareness, the diagnosis of Munchausen syndrome by proxy is controversial. In our opinion, an adolescent can manipulate the situation and simulate a disease, as can an adult, and may therefore be suspected of suffering from Munchausen syndrome. As this is a psychiatric diagnosis that usually applies to adults, there is currently no suitable diagnosis for a patient such as ours.

We hypothesized that this patient presented a factitious disorder or Munchausen syndrome, although confirmation requires psychiatric evaluation of the minor and his parents. This evaluation was not carried out in our center, since the confirmation of β-adrenergic agonist abuse was obtained after discharge of the patient.

## CONCLUSION

Severe hypokalemia secondary to chronic hidden abuse of β-adrenergic agonists in children has infrequently been reported in the literature. Medical management must focus on the severity of the patient’s symptoms. In the presence of an atypical presentation or an incoherent clinical history, the diagnostic workup should consider the possibility of a factitious disorder, the diagnosis of which will avoid costly and invasive diagnostic tests and unnecessary treatments. Adolescents can suffer a Munchausen syndrome in the same way as adults.
